# The emerging role of E3 ubiquitin ligase RNF213 as an antimicrobial host determinant

**DOI:** 10.3389/fcimb.2023.1205355

**Published:** 2023-08-15

**Authors:** Yulu Zhang, Yupei Yuan, Lu Jiang, Yihan Liu, Leiliang Zhang

**Affiliations:** ^1^ Department of Clinical Laboratory Medicine, The First Affiliated Hospital of Shandong First Medical University and Shandong Provincial Qianfoshan Hospital, Jinan, Shandong, China; ^2^ Department of Pathogen Biology, School of Clinical and Basic Medical Sciences, Shandong First Medical University and Shandong Academy of Medical Sciences, Jinan, Shandong, China; ^3^ Medical Science and Technology Innovation Center, Shandong First Medical University and Shandong Academy of Medical Sciences, Jinan, Shandong, China

**Keywords:** RNF213, antimicrobial infection, ubiquitination, lipid droplets, cell autonomous immunity

## Abstract

Ring finger protein 213 (RNF213) is a large E3 ubiquitin ligase with a molecular weight of 591 kDa that is associated with moyamoya disease, a rare cerebrovascular disease. It is located in the cytosol and perinuclear space. Missense mutations in this gene have been found to be more prevalent in patients with moyamoya disease compared with that in healthy individuals. Understanding the molecular function of RNF213 could provide insights into moyamoya disease. RNF213 contains a C3HC4-type RING finger domain with an E3 ubiquitin ligase domain and six AAA+ adenosine triphosphatase (ATPase) domains. It is the only known protein with both AAA+ ATPase and ubiquitin ligase activities. Recent studies have highlighted the role of RNF213 in fighting against microbial infections, including viruses, parasites, bacteria, and chlamydiae. This review aims to summarize the recent research progress on the mechanisms of RNF213 in pathogenic infections, which will aid researchers in understanding the antimicrobial role of RNF213.

## 1 Introduction

### 1.1 RNF213 and molecular structure

Ring finger protein 213 (RNF213) is a 591-kDa E3 ubiquitin ligase in humans that is also referred to as Mysterin (Moyamoya steno-occlusive disease–associated AAA+ and RING finger protein). Although RNF213 has been widely studied as a cause of moyamoya disease (MMD), it also plays important roles in other cellular processes and has been implicated in the immune response to pathogenic infections (Pollaci et al., 2022). The size of RNF213 is a challenge for laboratory manipulation, as it consists of 5,207 amino acids. As a result, studying the protein is difficult, and further research is required to gain a more comprehensive understanding of its structure and chemical activity, which is essential to fully comprehend its functions.

**Figure 1:**
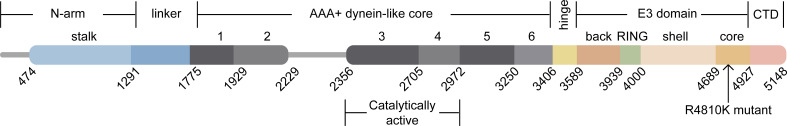
Domain architecture of RNF213. The N-arm (residues 1–1,290) is connected by a linker domain (1,291–1,774) to the six AAA+ units (1,775–3,405). Two of these AAA+ units are catalytically active, and the other four are inactive, comprising the dynein-like core. The central region is composed of a hinge domain (3,406–3,588) that connects the AAA+ core to the E3 domain (3,589–4,926). The ubiquitin ligase activity of RNF213 is expected to depend on the E3-RING domain (3,940–3,999). The last CTD is located at the edge of the molecule (4,927–5,148).

Ahel et al. employed Cryo-Electron Microscopy (Cryo-EM) analysis to obtain a detailed structure of the full-length mouse RNF213, which has a molecular weight of 584 kDa and consists of 5,148–amino acid residues (Ahel et al., 2020). This structure closely resembles the human homolog protein. The mouse RNF213 protein is composed of six ATPase structural domains, an E3 ubiquitin ligase domain, and a RING domain (Pollaci et al., 2022). The N-arm of the RNF213 protein (residues 1–1,290) is connected by a linker domain (1,291–1,774) to the six AAA+ units (1,775–3,405). The dynein-like core, which is responsible for the ATPase activity of RNF213, is composed of two catalytically active and four inactive AAA+ structural domains. The central region of the protein consists of a hinge domain (3,406–3,588) that links the AAA+ core to the E3 domain (3,589–4,926). The E3-RING domain (3,940–3,999), which is located on top of the E3 scaffold made up of the E3-back, E3-shell, and E3-core domains, is responsible for the ubiquitin ligase activity of RNF213. The last C-terminal domain is located at the edge of the protein (**Figure 1**). Thus, RNF213 is capable of both a dynein-like core function and a unique ubiquitin transfer function (Ahel et al., 2020). As a giant cytosolic protein, RNF213 is capable of forming a ring-shaped homo-oligomer inside the cell, and a portion of RNF213 diffuses in a monomeric way (Morito et al., 2014). In addition to its RING-mediated ubiquitin ligase activity, RNF213 can also promote ubiquitin transfer through a transthiolation reaction, which is a form of ubiquitination that does not depend on the RING structure. What sets RNF213 apart is that it is the only known protein to possess both AAA+ ATPase and ubiquitin ligase activities. The distinctive structure of RNF213 suggests that it has multiple functions.

### 1.2 RNF213 and vasculopathy

As the first susceptibility gene to be identified for MMD, RNF213 has been extensively studied in the context of angiopathy. MMD is a rare cause of stroke that is characterized by progressive stenosis of the terminal and compensatory capillary collateral branches of the internal carotid artery, which can be observed radiologically (Ihara et al., 2022). The *RNF213* gene is encoded by an ORF that is 15,624 bp long and located on chromosome 17q25.3, with Untranslated Regions (UTRs) of 5,431 bp in length at the 5′ and 3′ ends. In 2011, two independent Japanese studies found that the p.R4810K variant of *RNF213* was found to increase the risk of MMD by over 100-fold, based on analyses of patient families (Kamada et al., 2011; Liu et al., 2011). Missense mutations in this gene are significantly more common in patients with MMD than in the general population, with the *RNF213* R4810K mutant being a particularly prevalent sequence variant (also called p.R4810K, c.14429G>A, rs112735431). Subsequently, numerous follow-up studies have investigated the association between p.R4810K and MMD in East Asian populations. Comprehending the molecular function of RNF213 could significantly enhance our understanding of MMD (Asselman et al., 2022).

MMD is more likely to develop in genetically predisposed individuals (Liu et al., 2011; Takamatsu et al., 2017). However, non-p.R4810K rare missense variants have been found to be significantly associated with MMD in Caucasian patients, with the variants preferentially clustering in a C-terminal hotspot that encompasses the RING-finger domain of RNF213 (Guey et al., 2017). This suggests that there may be other genetic risk factors for MMD beyond the Asian *RNF213* p.R4810K variant, such as p.E4950D and p.A5021V (Liao et al., 2017). In addition, several studies have suggested that the p.R4810K variant on *RNF213* is associated with non-MMD intracranial major artery stenosis/occlusion (non-MMD ICASO) (Cheng et al., 2019). Kobayashi and colleagues have conducted *in vitro* and *in vivo* experiments to obtain biochemical and functional characterizations of p.R4810K in angiogenesis, demonstrating that the upregulation of *RNF213* can be induced by inflammatory signals and that the p.R4810K polymorphism leads to a decreased tube-forming ability in response to environmental stimuli (Kobayashi et al., 2015). There is growing evidence that *RNF213* and its mutants are linked to vasculopathy with a range of clinical presentations that include MMD as well as other intracranial and systemic vasculopathies (Hiraide et al., 2022). Although the connection between *RNF213* mutations and vascular disorders has been highlighted, recent research suggests that the ubiquitination activity of *RNF213* plays an essential role in host defense against microbial infections.

### 1.3 RNF213 and ubiquitin ligase activity

Ubiquitin (Ub) is a 76–amino acid protein that was first discovered by Dr. Gideon Goldstein in 1975 and is widely expressed in all eukaryotes (Goldstein et al., 1975). The total protein sequence of ubiquitin contains seven lysine sites (K6, K11, K27, K33, K48, and K63), a methionine site located at the N-terminal (Met1, also called M1 or linear), and a glycine site located at the C-terminal (G76) (Wilkinson and Audhya, 1981). The linear polyubiquitin chain, also known as M1 linkage, is assembled on proteins by the linear ubiquitin chain assembly complex (LUBAC), which is crucial in various signaling pathways (Rieser et al., 2013). RNF213, as an ubiquitin ligase, plays an essential role in generating the ubiquitin coat required for its unique structure (Zheng and Shabek, 2017). The process of ubiquitination involves the catalytic actions of ubiquitin-activating enzymes (E1), ubiquitin-conjugating enzymes (E2), and ubiquitin-protein ligases (E3). The specific biological functions of ubiquitin can be dictated by individual E2 enzymes, as the interaction between E2 and E3 determines the ultimate substrates that will be covalently bound by the E2 enzyme (Winkler and Timmers, 2005). Through yeast two-hybrid screening using a fragment containing the RING domain of RNF213 as bait, researchers identified UBC13 (UBE2N) as an E2 ubiquitin-conjugating enzyme for RNF213 E3 ubiquitin ligase (Habu and Harada, 2021). Further analysis of the ubiquitin chain on RNF213 revealed that RNF213 undergoes autoubiquitination primarily in the form of K63-linked chains, rather than K48-linked chains. This demonstrates that RNF213 functions as a K63-linked E3 ubiquitin ligase, and UBC13 plays a crucial role in mediating RNF213-dependent ubiquitination. Interestingly, RNF213 has the ability to form various types of ubiquitin chains, including M1 (Otten et al., 2021), K11 (Bhardwaj et al., 2022), K48 (Tian et al., 2023), and K63 (Habu and Harada, 2021), which may vary depending on the specific pathogens involved.

### 1.4 RNF213 and lipid droplets

The relationship between RNF213 and lipid droplets (LDs) is gradually becoming clearer. LDs consist of a neutral lipid core of triglycerides and sterol esters that are surrounded by a monolayer of phospholipids, which have long been deemed as solely neutral lipid storage compartments in cells. As cell-autonomous organelles, LDs possess a protein-mediated antimicrobial capacity, and RNF213 has been found on LDs (Bosch et al., 2020). In addition, LDs play fundamental roles in the regulation of inflammation and immunity (Monson et al., 2021). Certain pathogens, including bacteria, parasites, and viruses, induce LD accumulation, leading to enhanced immune responses to infection (Monson et al., 2021; de Almeida et al., 2023).

However, lipid accumulation can interfere with normal cellular and tissue functions, potentially causing lipotoxicity and various metabolic diseases (Brookheart et al., 2009). Palmitate, in particular, is toxic to cells, as high concentrations can induce apoptosis (Paumen et al., 1997). Piccolis and colleagues found that RNF213 depletion reduced palmitate-mediated cytotoxicity by approximately 50%, and knockdown of RNF213 attenuated the cell death induced by palmitate in hepatocellular carcinoma-derived cell line 2 (HepG2) cells and suspended mammary 159 pleural effusion (SUM159) cells (Piccolis et al., 2019). RNF213 is clearly a modulator of lipotoxicity from saturated fatty acids through a mechanism that affects the ability of cells to store lipids in LDs. Organisms require the nuclear transcription factor-kappa B (NF-κB) signaling pathway to initiate the inflammatory response when infected. Of note, the authors also noted that downstream of lipoxicity and RNF213 depletion have an impact on endoplasmic reticulum (ER) stress and NF-KB signaling, suggesting a link between RNF213 and the inflammatory response (Piccolis et al., 2019). However, the exact molecular mechanism remains to be investigated.

Moreover, Sugihara and colleagues reported the first evidence that RNF213 could physically interact with LDs (Sugihara et al., 2019). Overexpression of *RNF213* resulted in a notable increase in both the quantity and size of LDs within cells. Conversely, knockout or knockdown of *RNF213* resulted in a significant reduction in LDs abundance. These findings strongly suggest that *RNF213* plays a role in lipid storage *in vivo*. Consistently, *RNF213*-knockout (*RNF213*-KO) cells showed markedly increased adipose triglyceride lipase (ATGL) attached to LDs. Despite not discovering a physical interaction between RNF213 and the rate-limiting lipase ATGL, researchers have speculated that RNF213 may affect a putative anchoring protein through its AAA+ activity. By blocking the influx of ATGL into LDs and eliminating the ATGL in LDs, they reduced lipolysis and increased fat storage, thus regulating the formation of LDs (Sugihara et al., 2019). However, it was not clear whether monomeric or oligomeric RNF213 associates with LDs until recently. Indeed, some researchers have found that RNF213 oligomerization is associated with LDs in cells treated with type-1 interferon (IFN) (Thery et al., 2021). These findings indicate a deep relationship between RNF213 and LDs, which might be important for the antimicrobial infection function of RNF213.

## 2 Antiviral role of RNF213

Although researchers initially focused on studying the function of RNF213 in MMD, Echizenya et al. demonstrated that RNF213 could alleviate severe headaches in patients with enterovirus-induced hand, foot, and mouth disease. They revealed that RNF213 played an important role in cerebrovascular diseases caused by viral infections, thus beginning to unveil the link between RNF213 and viruses (Echizenya et al., 2020). However, the first evidence of a direct antiviral role of RNF213 was revealed by Houzelstein and colleagues (Houzelstein et al., 2021). Rift valley fever (RVF) is an acute viral zoonotic disease caused by RVF virus (RVFV) and transmitted by mosquito vectors or by contact. To demonstrate the antiviral role of RNF213, Houzelstein and colleagues generated *RNF213*-deficient mice using the clustered regularly interspaced short palindromic repeats (CRISPR)–CRISPR-associated 9 (Cas9) system. *RNF213*-deficient mice were found to be more susceptible to RVFV, whereas mice overexpressing *RNF213 in vivo* showed increased resistance to RVFV and exhibited reduced symptoms of infection compared with controls (Houzelstein et al., 2021). In addition, the expression of *RNF213* was significantly upregulated in experimental animals, such as chickens and ducks, upon injection with highly pathogenic strains of avian influenza (Pirbaluty et al., 2022). Together, these findings strongly indicate that RNF213 functions as an antiviral protein, playing a critical role in safeguarding against viral infections.

**Figure 2:**
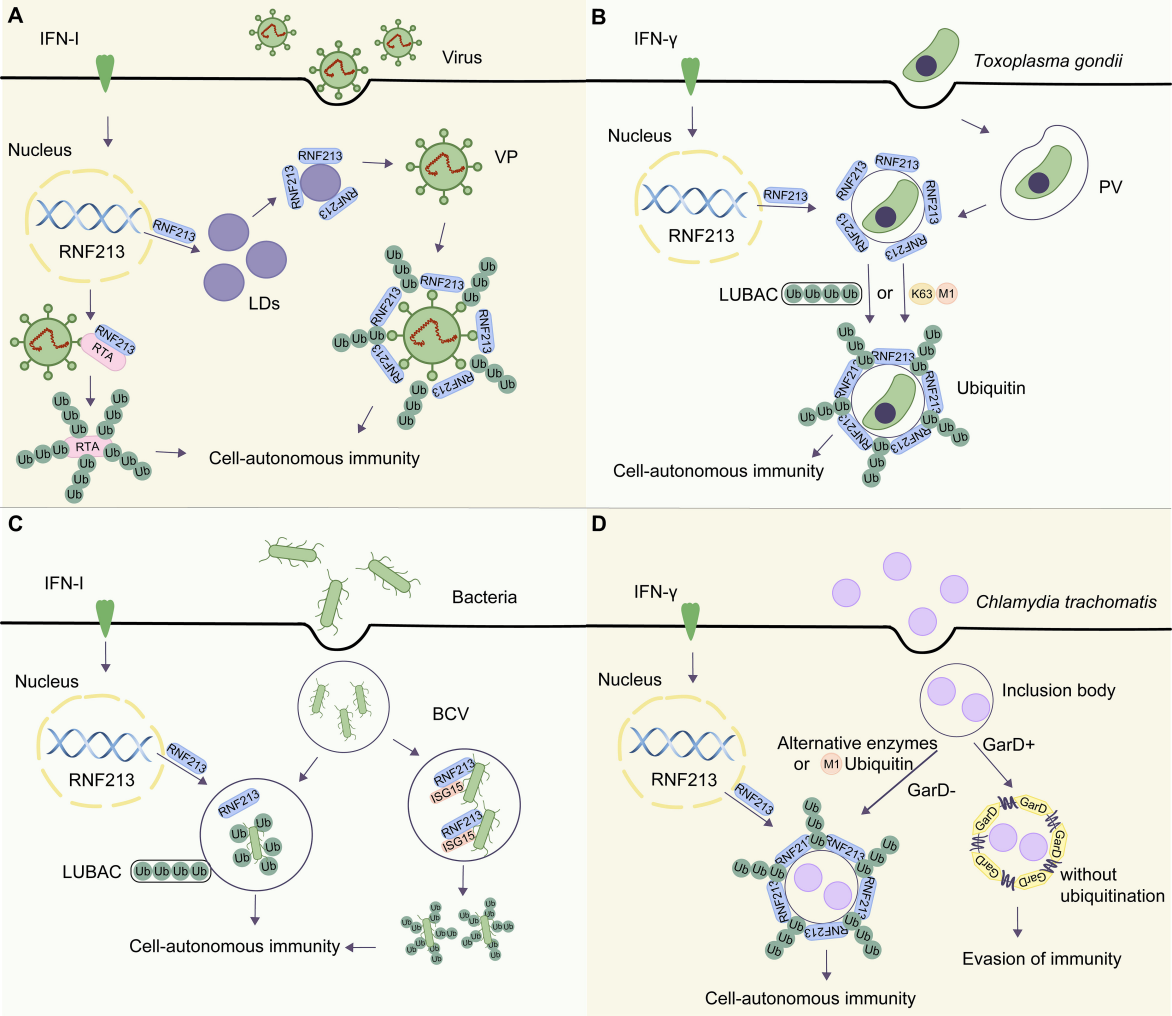
The roles of RNF213 in different microorganisms. **(A)** Upon HSV-1 infection, RNF213 targets LDs and is involved in the ubiquitination of viral particles (VPs) mediated by LDs. In KSHV infection, RNF213 acts as an E3 ubiquitin ligase to promote K48-linked polyubiquitination of protein RTA. This downregulates RTA and attenuates its function in initiating downstream replication and transcription. **(B)** Upon *Toxoplasma gondii* infection, RNF213 is translocated to the parasitophorous vacuole (PV). On one hand, RNF213 relies on the LUBAC to recruit ubiquitin adapter proteins and initiate PV ubiquitination. On the other hand, PV can be directly modified by M1-linked and K63-linked ubiquitin to initiate autophagy. **(C)** *Salmonella* enters the host cell via receptor-mediated endocytosis and forms a bacteria-containing vesicle (BCV). RNF213-mediated LPS ubiquitination can recruit the E3 ligase LUBAC, which catalyzes the formation of linear ubiquitin chains in the membrane of bacterial cells recognized by the organism. Finally, the host initiates cellular immune signaling and allogeneic phagocytosis. In *Listeria monocytogenes*, a Gram-positive bacterium that lacks LPS, RNF213, acts as an ISG15 binding protein that localizes to the bacterial surface and initiates ubiquitination. **(D)** In GarD-deficient chlamydial inclusion bodies, RNF213 is necessary for the formation of M1-ubiquitin chains to exert anti-Chlamydia activity. However, in the presence of GarD, RNF213 cannot mediate the ubiquitination of inclusion bodies, leading to evasion of immunity.

Extensive research studies have been conducted recently on the antiviral activity of RNF213, unveiling the links between ISGylated proteins, RNF213, and antiviral immunity. IFN-stimulated gene 15 (*ISG15*) protein is a ubiquitin-like protein that can be strongly induced by viral (Yuan and Krug, 2001; Jurczyszak et al., 2022), bacterial infections (Millman et al., 2022), and IFNs (Der et al., 1998; Hermann and Bogunovic, 2017). ISG15 has been implicated as a central player in the host antiviral response (Perng and Lenschow, 2018). Thery and colleagues used the Virotrap approach, which captures protein complexes within virus-like particles (VLPs) that bud from mammalian cells, to identify noncovalent interaction partners of ISG15. They confirmed the presence of RNF213 in the ISG15 VLPs and verified RNF213 as an ISG15-binding protein. Further experiments showed that RNF213 specifically associated with ISG15, defining RNF213 as a sensor for ISGylated protein. The association of RNF213 with LDs was increased upon IFN treatment, accompanied by a notably enhanced appearance of a smear of ISGylated proteins. Mechanistically, they confirmed that IFN-induced RNF213 associated with ISGylated proteins on the surface of LDs *in vitro* (Thery et al., 2021). Furthermore, Thery and colleagues used herpes simplex virus (HSV-1), respiratory syncytial virus (RSV), and coxsackievirus (CVB3), all of which are ISG15-sensitive viruses. Knockdown of *RNF213* in HeLa cells increased the genome replication of the virus and the expression level of viral proteins. However, it is unclear whether RNF213 binds ISGylated proteins on LDs in the three viruses resembling *the vitro* experiment and requires further verification (Thery et al., 2021). The work of Thery and colleagues suggests that the RNF213–ISG15 association in LDs plays an important role in the antiviral process (**Figure 2A**). However, the restriction of HSV-1 by RNF213 does not require ISG15, whereas the protective effect of RNF213 against an intracellular bacterial pathogen *Listeria monocytogenes* was dependent on ISG15, indicating different mechanisms for the antiviral and antibacterial effects.

Kaposi’s sarcoma-associated herpesvirus (KSHV), a type of *γ-herpesvirus* linked to several human malignancies, has not been shown to be efficiently inhibited by IFN in its lytic replication. Researchers constructed an expression library of ISGs and used murine γ-herpesvirus 68 (MHV-68) as a model virus for KSHV study. They identified several ISGs that could inhibit the replication of MHV-68 (Tian et al., 2023). Of these ISGs, RNF213 significantly suppressed the early gene transcription and genome replication of the virus. Mechanistically, RNF213 acted as an E3 ubiquitin ligase to promote K48-linked polyubiquitination of a viral protein called RTA (replication and transcription activator), which subsequently led to RTA degradation via the proteasome-dependent pathway. KSHV infection undergoes a switch from the latent phase to lytic replication governed by RTA, known as the “molecular switch.” The downregulated RTA is subsequently attenuated in its function in initiating downstream replication and transcription (**Figure 2A**). Intriguingly, RNF213 degrades RTA protein via the proteasomal pathway, rather than the autophagy-lysosome pathway. In summary, this study highlights the role of RNF213 in inhibiting KSHV and provides a novel idea for effective prevention and control of viral infection.

The immune system plays a crucial role in the antiviral response. When viruses invade the body, antigen-presenting cells (APCs) uptake antigens and present them to T cells, which then initiate a cascade of immune responses. To further explore the function of RNF213, researchers generated *RNF213*-KO and *RNF213*-knockin mice with single-nucleotide insertions corresponding to mutations found in patients with MMD. Mice with dysregulated expression of the *RNF213* gene exhibited reduced dendritic cell development, antigen processing, and presentation functions (Tashiro et al., 2021). Hence, RNF213 might aid in eliminating viruses through T cells, thereby achieving antiviral effects. However, the specific mechanism by which RNF213 contributes to the reduced function of APCs requires further investigation.

## 3 Anti-parasitic role of RNF213

*Toxoplasma gondii* (TO) is a parasite that is capable of causing zoonotic parasitic diseases. Previous studies have shown that immunity-related guanosine triphosphatases (GTPases) (IRG) and guanylate-binding protein (GBP) work cooperatively in the cell-autonomous immune response to *Toxoplasma* in mouse cells and can promote each other’s recruitment to parasitophorous vacuoles (PVs) (Bhushan et al., 2020; Frickel and Hunter, 2021). However, the mechanism by which IRGs and GBPs cooperatively detect and destroy PVs is still under exploration. Researchers have found that IFN-γ–primed host cells prompt IRG-dependent association of *Toxoplasma*-containing vacuoles with ubiquitin through the regulated translocation of the E3 ubiquitin ligase tumor necrosis factor (TNF) receptor–associated factor 6 (TRAF6) (Haldar et al., 2015). In addition, different members of the TRAF family can localize to *Toxoplasma* PVs and enhance the cell-autonomous immunity to *Toxoplasma* by ubiquitination in IFN-γ–primed cells (Mukhopadhyay et al., 2020). Moreover, it has been found that the IFN-γ–mediated growth restriction of *Toxoplasma gondii* depends on the core components of the autophagy pathway but not on the initiation or degradative steps of autophagy. ISG15, which is upregulated by IFN-γ along with other members of the ISGylation pathway, interacts with members of the autophagy-related gene (ATG). Although ISG15 does not affect the ubiquitination of the PV, it plays a crucial role in recruiting autophagy mediators such as p62 (SQSTM1), NDP52 (CALCOCO2), and Microtubule-associated protein light chain 3 (LC3) to the PV, leading to growth restriction of *Toxoplasma gondii* by ATG. Thus, ISG15 serves as a vital molecular link between the ATG and IFN-γ signaling pathways, enabling cell-autonomous defense against parasites in human cells (Bhushan et al., 2020).

Hernandez and colleagues further investigated the anti-parasitic potency of RNF213 and found that IFN-γ–stimulated cells failed to significantly reduce *Toxoplasma* burden in *RNF213*-KO cells compared with that in wild-type (WT) cells. The loss of cell-autonomous immunity in *RNF213*-deficient cells was confirmed by plaque assays and measuring relative light units emitted by luminescent strains. These data verified that RNF213 executes anti-parasitic activity in IFN-γ–stimulated A549 cells and characterized RNF213 as a potent executioner of human anti-parasitic host defense. Furthermore, the researchers demonstrated that IFN-γ induced RNF213 to translocate to the surface of PVs with LUBAC. Subsequently, the host cell could initiate the degradation of intracellular substances through a noncanonical autophagy-related process. Interestingly, linear ubiquitination of PVs also occurred in cells lacking essential components of LUBAC, suggesting that LUBAC is dispensable for *Toxoplasma* PVs ubiquitination and cell-autonomous host defense. It is a really notable discovery because LUBAC was the only known enzyme to catalyze M1-ubiquitin chain, suggesting that other E3 ubiquitin ligases catalyzing M1 chain might exist in human but are yet to be discovered. Moreover, RNF213 could recruit adaptor proteins of autophagy, such as p62, TAX1BP1 (Tax1 binding protein 1), NDP52 and optineurin (OPTN), to ubiquitinated *Toxoplasma* PVs, which were modified by the linear-linked (M1) ubiquitin and K63-linked ubiquitin. Therefore, the host was able to recognize the ubiquitin-labeled PVs and to initiate the host immune defense against *Toxoplasma gondii* infection (Hernandez et al., 2022) (**Figure 2B**). RNF213 mediates IFN-γ–dependent M1- and K63-linked ubiquitination of the *Toxoplasma* PVs to control pathogen replication, which is likely a shared pattern of pathogenesis between distinct intracellular pathogens (Gilliland and Olive, 2022). However, it is unclear whether RNF213 catalyzes M1- or K63-ubiquitin chain directly *in vivo*. Likely, RNF213 conjugates the first ubiquitin repeat on PVs, whereas other E3 ubiquitin ligases conjugate secondary ubiquitin repeats, which remains to be demonstrated (Otten et al., 2021). In the future, it would be valuable for researchers to investigate how RNF213 exerts anti-parasitic effects on other parasites and whether RNF213 acts by similar mechanisms with other types of microorganisms. This could provide insights into the potential broad-spectrum antimicrobial activity of RNF213 and its potential as a therapeutic target for infectious diseases. In addition, further studies could explore the potential involvement of RNF213 in other aspects of the immune response and its potential role in the development of immune-related disorders.

## 4 Anti-bacterial role of RNF213

Emerging evidence suggests that RNF213 plays a significant role in anti-bacterial immunity, and, unexpectedly, the substrates of its ubiquitination activity extend beyond the proteome. Although lysine ubiquitination is traditionally considered canonical, there is a growing recognition of atypical non-lysine ubiquitination, which is gradually being established as an important regulatory mechanism (Kelsall, 2022). Otten et al. reported that RNF213 can recognize and ubiquitinate lipid A, a component of the outer wall of Gram-negative bacteria, breaking the dogma that only proteins can be ubiquitinated substrates (Otten et al., 2021). To detect ubiquitin on the bacterial surface, they carried out immunoblotting with FK2, an antibody specific for conjugated ubiquitin, and revealed a ubiquitin smear above 50 kDa in WT *Salmonella* (*S.*) Typhimurium. However, *S.* Typhimurium *Drfc*, lacking the O-antigen polymerase required for smooth LPS synthesis, carried ubiquitinated products of lower molecular weight. They further demonstrated the ubiquitination of LPS (Ub-LPS) *in vitro*. To identify the enzyme that could generate Ub-LPS, they fractionated HeLa cell lysates by sequential ammonium sulfate precipitation, hydrophobic interaction chromatography, gel-filtration, and ion exchange chromatography, followed by mass spectrometry. RNF213 was found to be the only protein whose peptide counts matched the LPS-ubiquitinated activity. The researchers attenuated *RNF213* expression by small interfering RNA (siRNA) or CRISPR-based knockout in human and mouse cells to explore whether RNF213 is required for the Ub-LPS and the functional importance of RNF213 for cell-autonomous immunity. Upon *RNF213* downregulation, the presence of Ub-LPS in the Gram-negative bacteria *S.* Typhimurium was reduced, indicating an essential role for human and murine RNF213 in LPS ubiquitination on the bacteria (Otten et al., 2021). Subsequently, the host cells initiated pathogenic autophagic degradation against bacterial invasion (**Figure 2C**). *In vitro* studies have demonstrated that purified RNF213 can ubiquitinate LPS in a canonical manner, necessitating the presence of adenosine triphosphate (ATP), E1, and E2 enzymes. Unexpectedly, the RING domain of RNF213 is not required for its intrinsic E3 ligase activity or its Ub-LPS (Ahel et al., 2020; Otten et al., 2021). Collectively, it can be concluded that RNF213 has the ability to ubiquitinate LPS from Gram-negative bacteria invading the cytoplasmic matrix *in vivo*, and the process of ubiquitination is dependent on the dynein-like core of RNF213. The ability of RNF213 to ubiquitinate LPS breaks the previous notion that only proteins can be ubiquitinated substrates and provides a new research direction to study the antimicrobial function of RNF213.

It is worth noting that the E3 ligase LUBAC catalyzes the formation of Met1-linked linear ubiquitin chains, which have been demonstrated to play a pivotal role in cellular autonomous immunity (Noad et al., 2017). Otten et al. knocked out *RNF213* and found that *RNF213*-deficient cells failed to recruit LUBAC and Nemo (the M1-specific adaptor) and that *RNF213* deficiency could hamper M1-linked ubiquitin chains to the bacterial ubiquitin coat. The research also confirmed that *RNF213*-KO cells lost the capability to recruit autophagy cargo receptors associated with ubiquitin (Otten et al., 2021). Therefore, when host cells lack the *RNF213* gene or contain mutations in the catalytic zinc-binding domain of the protein, the cells fail to recruit LUBAC and to accumulate Met1-linked ubiquitin chains on the bacterial cell surface. On the basis of this, the researchers demonstrated that lipid A can be ubiquitinated, leading to the removal of bacteria by immune signaling and xenophagy. *Salmonella* enters the host cell via receptor-mediated endocytosis and forms a bacteria-containing vesicle (BCV). The vesicle ruptures, and the bacteria are exposed to the ubiquitination system mediated by RNF213. RNF213-mediated LPS ubiquitination can recruit the E3 ligase LUBAC, which catalyzes the formation of linear ubiquitin chains in the membrane of bacterial cells recognized by the organism. Finally, the host initiates cellular immune signaling and allogeneic phagocytosis (**Figure 2C**). More importantly, the mechanisms underlying how the dynein-like ATPase core and the RING finger contribute to RNF213 function and how LPS is recognized remain to be elucidated. Answers to these questions and intensive research may facilitate a profound understanding of the anti-bacterial potency of RNF213 and offer insights into ubiquitination-dependent cell-autonomous defense against cytosolic bacterial invaders (Peng et al., 2021).

RNF213 has been reported to counteract infection not only with *S.* Typhimurium but also with *Listeria monocytogenes*, a Gram-positive bacterium that lacks LPS, implying functional diversity of RNF213 (Martina et al., 2021; Thery et al., 2021). By knocking down *RNF213*, Thery and colleagues found a significant increase in intracellular *Listeria* bacterial load, similar to knockdown of *ISG15*. Moreover, in *ISG15* knockout cells, even overexpressed *RNF213* could not play a protective role, indicating that the antibacterial activity of RNF213 requires ISG15. In contrast, unlike the antiviral effect, the restriction of HSV-1 by RNF213 does not require ISG15 (Thery et al., 2021). They further showed that RNF213 acted as a binding protein for ISG15 and localized at the bacterial surface to exert an antibacterial effect (**Figure 2C**), whereas other researchers have observed that *ISG15* expression could restrict *Listeria* infection *in vitro* and *in vivo* (Radoshevich et al., 2015). The study by Thery et al. suggests that RNF213 can make a significant difference in cellular autonomous immunity and emphasizes the role of the RNF213-ISG15 association in the antibacterial effect. In addition, RNF213 can also regulate the transcription of *dimethylarginine dimethylaminohydrolase 1* (DDAH1) and the production of nitric oxide to facilitate anti-listerial effects (Martina et al., 2021). Collectively, RNF213 can induce cellular autophagy and stimulate the function of cellular autonomous immunity. This specific mechanism of the antimicrobial function of RNF213 is gradually being redefined as an immune signaling pathway. The antimicrobial activity of RNF213 seems to be specifically targeted toward intracellular bacterial pathogens.

## 5 Anti-Chlamydia role of RNF213

In addition to studies investigating the antimicrobial infection of RNF213 function, researchers have found that there are also corresponding anti-ubiquitination factors in microorganisms to resist cell-autonomous immunity produced by RNF213, which opens an exciting chapter in pathogen immune evasion (Walsh et al., 2022). By genetic screening using an arrayed library of mutagenized chlamydiae, researchers identified the chlamydial inclusion body membrane protein gamma resistance determinant (GarD; also called CTL0390) as a chlamydial effector. Further analysis revealed that the structure of GarD is similar to that of other type III secreted inclusion body membrane proteins, and it is inserted into the inclusion bodies during infection. Thereafter, the inclusion bodies are not tagged by IFN-γ–dependent ubiquitin, which averts the association of lysosomal-associated membrane protein 1 with the inclusion bodies, allowing *Chlamydia* (*C*.) *trachomatis* to evade immune detection and destruction driven by RNF213 (Walsh et al., 2022). Subsequently, *GarD*::GII inclusion bodies (GarD−) were formed with the insertional inactivation mutants of *GarD*, which could be modified by linear ubiquitin with RNF213 in IFN-γ–induced human epithelial cells. Therefore, the ubiquitin E3 ligase RNF213 was identified as a candidate anti-chlamydial protein. However, the WT (GarD+) secretes the GarD protein to stabilize the pathogen surrounding membranes and remains devoid of ubiquitin in IFN-γ–primed cells to safeguard *C. trachomatis* from ubiquitination and associated cell-autonomous immunity. In *GarD*-deficient *C. trachomatis* inclusion bodies, RNF213 is necessary for the formation of the M1-ubiquitin chain, but it is unclear whether RNF213 directly catalyzes the formation of the M1-ubiquitin chain. Interestingly, the LUBAC complex is redundant for this process. This study proposes that other E3 ligases mediating M1-ubiquitin chain types might exist as an alternative to the LUBAC complex (**Figure 2D**).

Furthermore, Walsh and colleagues confirmed that GarD acts as a cis-acting factor to execute its anti-ubiquitination, directly at the inclusion membrane on which it resides. To capitalize on the ability of *C. trachomatis* inclusions to fuse with each other, they insertionally inactivated *IncA*, a chromosomal gene encoding a protein required for homotypic fusion of chlamydial inclusion bodies. Co-infections of *GarD*::GII with WT *C. trachomatis* resulted in fused inclusion bodies containing a mix of WT and *GarD* mutant and a reduction in inclusion ubiquitination. However, inclusion bodies formed by *GarD*::GII in cells co-infected with fusion-deficient *IncA* continued to be ubiquitinated at high frequency. Therefore, they defined GarD as an RNF213 antagonist that is necessary for *C. trachomatis* to proliferate during IFN-γ–stimulated cell-autonomous immunity in the host cell. Of note, it is likely that RNF213 catalyzes the attachment of the first ubiquitin on nonproteinaceous substrates on the inclusion bodies, which is followed by the formation of M1-type ubiquitin chains by alternative enzymes instead of the LUBAC complex. Collectively, this research suggests that the antimicrobial function of RNF213 can be exerted only when the action of GarD is inhibited. This discovery could be leveraged to develop a novel therapeutic approach to treat infection with Chlamydia. It will be interesting and meaningful to identify the microbial virulence factors associated with RNF213 to resist host immunity in other microorganisms.

## 6 Conclusions and future prospects

Since the discovery of the RNF213 gene in 2011 (Kamada et al., 2011), its role in cardiovascular and cerebrovascular diseases has received a lot of attention due to the identification of *RNF213* as a susceptibility gene for MMD. In recent years, the novel function of RNF213 in the microbiology field has started to be unfolded. RNF213 plays an important role in combating viruses, parasites, bacteria, and chlamydiae, which are summarized in **Table 1**.

**Table 1:** Summary of RNF213 functions in different microorganisms.

Microorganisms		Roles of RNF213
Virus	Rift valley fever virus	RNF213 has the ability to inhibit viral infection (Houzelstein et al., 2021).
	Herpes simplex virus 1, Respiratory syncytial virus, Coxsackie virus B3	RNF213 associates with ISGylated proteins to target intracellular LDs, which maybe mediate the ubiquitination of RNF213 in viral particles (VP) and participate in cellular antiviral functions (Thery et al., 2021).
	γ-Herpesvirus	RNF213 promotes polyubiquitination modifications of protein RTA in a K48-linked manner, resulting in the reduction of early gene transcription and genome replication of the virus (Tian et al., 2023).
Parasites	*Toxoplasma gondii*	RNF213 can translocate to the surface of *Toxoplasma* PVs with LUBAC, leading to the initiation of autophagy by the host cells. In addition, RNF213 can recruit ubiquitin adaptor proteins and initiate the modification of PVs with both linear-linked ubiquitin and K63-linked ubiquitin (Hernandez et al., 2022).
Bacteria	*Salmonella* Typhimurium	RNF213 has been found to label the lipid A of bacteria by ubiquitination and subsequently initiate the pathogen autophagic degradation. Moreover, RNF213-mediated ubiquitination of LPS can recruit LUBAC in the host cell (Otten et al., 2021).
	*Listeria monocytogenes*	RNF213 functions as an ISG15 binding protein, which in turn induces cellular autophagy and stimulates cellular autonomic immunity (Thery et al., 2021).
Chlamydia	*Chlamydia trachomatis*	In the absence of GarD, inclusion bodies could be decorated by linear ubiquitin in IFN-γ–induced human epithelial cells with RNF213. However, when wild-type GarD was present, it could protect the inclusion bodies from cell-autonomous immunity (Walsh et al., 2022).

In the antiviral role of RNF213, researchers have linked the antiviral effects of RNF213 to LDs. RNF213 could bind ISG15 treated with IFN-I in human embryonic kidney 293T (HEK293T) cells, Henrietta Lacks (HeLa) cells, and tumor histiocyte-like monocyte 1 (THP-1) cells and associate with ISGylated proteins on LDs *in vitro*, suggesting that RNF213 is able to exert cellular antiviral activity by similar mechanisms (Thery et al., 2021). However, how RNF213 is recruited to the LDs? Additional research is necessary to ascertain the involvement of LDs in the ubiquitination process of RNF213 in host cells, and the connection between RNF213 and LDs should be explored further. Moreover, different types of polyubiquitin chains, including M1 and K11 chains, have been implicated in NF-κB activation (Iwai, 2012; Tokunaga, 2013). RNF213 might exert its role in infection with M1-ubiquitin chain involved in NF-κB activation. In KSHV infection, RNF213 promotes polyubiquitination modifications of viral protein RTA in K48-linked manner. This downregulates RTA and attenuates its function, resulting in the reduction of early gene transcription and genome replication of the virus (Tian et al., 2023). Similarly, how RNF213 recognize the RTA protein from the herpes virus? Does RNF213 colocalize with the virus or viral components like the co-localization with intracellular bacterial pathogens? Answers to these questions and intensive studies may facilitate a profound understanding of the antiviral functions of RNF213.

In the anti-parasitic role of RNF213, the mechanism focuses on the ubiquitination pathway in *Toxoplasma gondii* infection. RNF213 can translocate to the surface of PVs with or without LUBAC and execute cell-autonomous host defense. Whether other E3 ubiquitin ligases catalyzing M1-ubiquitin chains exist in human should be further investigated. In addition, RNF213 can recruit ubiquitin adaptor proteins and facilitate the modification of PVs with both M1- and K63-linked ubiquitin (Hernandez et al., 2022). However, it is unclear whether RNF213 catalyzes M1- or K63-ubiquitin chain directly. What the specific process of ubiquitination is like and how RNF213 and other E3 ubiquitin ligase conjugate the ubiquitin chains on PVs remain to be demonstrated. Moreover, whether similar or different mechanisms exist in other types of parasites needs to be further investigated.

In the anti-bacterial activity of RNF213, researchers have unveiled an initial set of mechanisms to combat Gram-positive and Gram-negative bacterial infections. Researchers have made a breakthrough in that the LPS of Gram-positive bacteria *Salmonella* can be ubiquitinated through K63-linked polyubiquitination or a new pathway involving LUBAC (Otten et al., 2021). Therefore, ubiquitin plays an important role in the host cell’s defense against bacterial infection. Although lysine as the substrate may still be considered canonical, it is becoming increasingly clear that ubiquitin can modify cysteine, serine, and threonine residues, as well as the N-terminal amino group of proteins (Kelsall, 2022). Because RNF213 conjugates to non-protein substrates such as the lipid A core in LPS, does RNF213 conjugate ubiquitin to host lipid, for instance, on LDs? This also provides researchers with a new perspective to study RNF213 ubiquitination and to focus on other kinds of interactors, such as lipid or sugar, which will improve the comprehensive understanding of the antiviral and anti-bacterial activities of RNF213. In addition, researchers have also revealed several RNF213-binding proteins. The interaction between RNF213 and ISG15 was discovered and demonstrated to be functionally important in the host infected by the Gram-negative bacteria *Listeria monocytogenes*. It is worth mentioning that RNF213 associates with ISG15 to fight against *Listeria* but not HSV-1. It suggests that the RNF213-ISG15 interaction differs between different microorganisms, indicating the deeper relationship between RNF213 and other interactors (Thery et al., 2021).

In *Chlamydia trachomatis* infection, important clues have successfully identified GarD as the first example of pathogen evasion within this previously unknown IFN-γ–dependent cell-autonomous immune pathway (Gilliland and Olive, 2022). In the absence of GarD, RNF213 could decorate inclusion bodies by linear ubiquitin. However, when WT GarD was present, it could protect the inclusion bodies from cell-autonomous immunity (Walsh et al., 2022). Are there other immune evasion mechanisms similar with GarD of *Chlamydia* in other pathogenic microorganisms? Further molecular mechanisms of cell-autonomous immunity caused by RNF213 and pathogenic immunity resistance should be studied more extensively.

In conclusion, as an antimicrobial host determinant, the emerging role of RNF213 in antimicrobial infections has been highlighted in many studies, especially in recent years. However, it needs more researchers to further investigate the mechanisms of antimicrobial functions of RNF213 in detail.

## Author contributions

LZ conceived the study. YZ wrote the first draft. YY, LJ, and LZ revised the manuscript. YZ and YL generated the Figures. All authors contributed to the article and approved the submitted version.

## References

[B1] Ahel J. Lehner A. Vogel A. Schleiffer A. Meinhart A. Haselbach D.. (2020). Moyamoya disease factor RNF213 is a giant E3 ligase with a dynein-like core and a distinct ubiquitin-transfer mechanism. Elife 9, e56185. doi: 10.7554/eLife.56185 32573437 PMC7311170

[B2] Asselman C. Hemelsoet D. Eggermont D. Dermaut B. Impens F. (2022). Moyamoya disease emerging as an immune-related angiopathy. Trends Mol. Med. 28 (11), 939–950. doi: 10.1016/j.molmed.2022.08.009 36115805

[B3] Bhardwaj A. Banh R. S. Zhang W. Sidhu S. S. Neel B. G. (2022). MMD-associated RNF213 SNPs encode dominant-negative alleles that globally impair ubiquitylation. Life Sci. Alliance 5 (5), e202000807. doi: 10.26508/lsa.202000807 35135845 PMC8831215

[B4] Bhushan J. Radke J. B. Perng Y. C. McAllaster M. Lenschow D. J. Virgin H. W.. (2020). ISG15 connects autophagy and IFN-γ-dependent control of toxoplasma gondii infection in human cells. mBio 11 (5), e00852-20. doi: 10.1128/mBio.00852-20 33024031 PMC7542356

[B5] Bosch M. Sánchez-Álvarez M. Fajardo A. Kapetanovic R. Steiner B. Dutra F.. (2020). MamMalian lipid droplets are innate immune hubs integrating cell metabolism and host defense. Science 370 (6514), eaay8085. doi: 10.1126/science.aay8085 33060333

[B6] Brookheart R. T. Michel C. I. Schaffer J. E. (2009). As a matter of fat. Cell Metab. 10 (1), 9–12. doi: 10.1016/j.cmet.2009.03.011 19583949 PMC2751821

[B7] Cheng W. Xue S. Wu F. Song X. Huang Q. Song H.. (2019). The clinical and vascular characteristics of RNF213 c.14576G>A variant-related intracranial major artery disease in China. Behav. Neurol. 2019, 7908392. doi: 10.1155/2019/7908392 30992731 PMC6434291

[B8] de Almeida P. E. Pereira de Sousa N. M. Rampinelli P. G. Silva R. V. S. Correa J. R. D'Avila H. (2023). Lipid droplets as multifunctional organelles related to the mechanism of evasion during mycobacterial infection. Front. Cell Infect. Microbiol. 13. doi: 10.3389/fcimb.2023.1102643 PMC9996354 36909724

[B9] Der S. D. Zhou A. Williams B. R. Silverman R. H. (1998). Identification of genes differentially regulated by interferon alpha, beta, or gamma using oligonucleotide arrays. Proc. Natl. Acad. Sci. U.S.A. 95 (26), 15623–15628. doi: 10.1073/pnas.95.26.15623 9861020 PMC28094

[B10] Echizenya I. Tokairin K. Kawabori M. Kazumata K. Houkin K. (2020). Reversible cerebral angiopathy after viral infection in a pediatric patient with genetic variant of RNF213. J. Stroke Cerebrovasc Dis. 29 (2), 104549. doi: 10.1016/j.jstrokecerebrovasdis.2019.104549 31818681

[B11] Frickel E. M. Hunter C. A. (2021). Lessons from Toxoplasma: Host responses that mediate parasite control and the microbial effectors that subvert them. J. Exp. Med. 218 (11), e20201314. doi: 10.1084/jem.20201314 34670268 PMC8532566

[B12] Gilliland H. N. Olive A. J. (2022). GarD-ing the pathogen-containing vacuole from destruction. Cell Host Microbe 30 (12), 1655–1657. doi: 10.1016/j.chom.2022.11.007 36521440

[B13] Goldstein G. Scheid M. Hammerling U. Schlesinger D. H. Niall H. D. Boyse E. A. (1975). Isolation of a polypeptide that has lymphocyte-differentiating properties and is probably represented universally in living cells. Proc. Natl. Acad. Sci. U.S.A. 72 (1), 11–15. doi: 10.1073/pnas.72.1.11 1078892 PMC432229

[B14] Guey S. Kraemer M. Hervé D. Ludwig T. Kossorotoff M. Bergametti F.. (2017). Rare RNF213 variants in the C-terminal region encompassing the RING-finger domain are associated with moyamoya angiopathy in Caucasians. Eur. J. Hum. Genet. 25 (8), 995–1003. doi: 10.1038/ejhg.2017.92 28635953 PMC5567158

[B15] Habu T. Harada K. H. (2021). UBC13 is an RNF213-associated E2 ubiquitin-conjugating enzyme, and Lysine 63-linked ubiquitination by the RNF213-UBC13 axis is responsible for angiogenic activity. FASEB Bioadv 3 (4), 243–258. doi: 10.1096/fba.2019-00092 33842849 PMC8019261

[B16] Haldar A. K. Foltz C. Finethy R. Piro A. S. Feeley E. M. Pilla-Moffett D. M.. (2015). Ubiquitin systems mark pathogen-containing vacuoles as targets for host defense by guanylate binding proteins. Proc. Natl. Acad. Sci. U.S.A. 112 (41), E5628–E5637. doi: 10.1073/pnas.1515966112 26417105 PMC4611635

[B17] Hermann M. Bogunovic D. (2017). ISG15: in sickness and in health. Trends Immunol. 38 (2), 79–93. doi: 10.1016/j.it.2016.11.001 27887993

[B18] Hernandez D. Walsh S. Saavedra Sanchez L. Dickinson M. S. Coers J. (2022). Interferon-inducible E3 ligase RNF213 facilitates host-protective linear and K63-linked ubiquitylation of toxoplasma gondii parasitophorous vacuoles. mBio 13 (5), e0188822. doi: 10.1128/mbio.01888-22 36154443 PMC9601232

[B19] Hiraide T. Suzuki H. Momoi M. Shinya Y. Fukuda K. Kosaki K.. (2022). RNF213-associated vascular disease: A concept unifying various vasculopathies. Life (Basel) 12 (4), 555. doi: 10.3390/life12040555 35455046 PMC9032981

[B20] Houzelstein D. Simon-Chazottes D. Batista L. Tokuda S. Langa Vives F. Flamand M.. (2021). The ring finger protein 213 gene (Rnf213) contributes to Rift Valley fever resistance in mice. Mamm Genome 32 (1), 30–37. doi: 10.1007/s00335-020-09856-y 33420513

[B21] Ihara M. Yamamoto Y. Hattori Y. Liu W. Kobayashi H. Ishiyama H.. (2022). Moyamoya disease: diagnosis and interventions. Lancet Neurol. 21 (8), 747–758. doi: 10.1016/s1474-4422(22)00165-x 35605621

[B22] Iwai K. (2012). Diverse ubiquitin signaling in NF-κB activation. Trends Cell Biol. 22 (7), 355–364. doi: 10.1016/j.tcb.2012.04.001 22543051

[B23] Jurczyszak D. Manganaro L. Buta S. Gruber C. Martin-Fernandez M. Taft J.. (2022). ISG15 deficiency restricts HIV-1 infection. PloS Pathog. 18 (3), e1010405. doi: 10.1371/journal.ppat.1010405 35333911 PMC8986114

[B24] Kamada F. Aoki Y. Narisawa A. Abe Y. Komatsuzaki S. Kikuchi A.. (2011). A genome-wide association study identifies RNF213 as the first Moyamoya disease gene. J. Hum. Genet. 56 (1), 34–40. doi: 10.1038/jhg.2010.132 21048783

[B25] Kelsall I. R. (2022). Non-lysine ubiquitylation: Doing things differently. Front. Mol. Biosci. 9. doi: 10.3389/fmolb.2022.1008175 PMC9527308 36200073

[B26] Kobayashi H. Matsuda Y. Hitomi T. Okuda H. Shioi H. Matsuda T.. (2015). Biochemical and functional characterization of RNF213 (Mysterin) R4810K, a susceptibility mutation of moyamoya disease, in angiogenesis *in vitro* and *in vivo* . J. Am. Heart Assoc. 4 (7), e002146. doi: 10.1161/jaha.115.002146 26126547 PMC4608092

[B27] Liao X. Deng J. Dai W. Zhang T. Yan J. (2017). Rare variants of RNF213 and moyamoya/non-moyamoya intracranial artery stenosis/occlusion disease risk: a meta-analysis and systematic review. Environ. Health Prev. Med. 22 (1), 75. doi: 10.1186/s12199-017-0680-1 29165161 PMC5667490

[B28] Liu W. Morito D. Takashima S. Mineharu Y. Kobayashi H. Hitomi T.. (2011). Identification of RNF213 as a susceptibility gene for moyamoya disease and its possible role in vascular development. PloS One 6 (7), e22542. doi: 10.1371/journal.pone.0022542 21799892 PMC3140517

[B29] Martina L. Asselman C. Thery F. Boucher K. Delhaye L. Maia T. M.. (2021). Proteome profiling of RNF213 depleted cells reveals nitric oxide regulator DDAH1 antilisterial activity. Front. Cell Infect. Microbiol. 11. doi: 10.3389/fcimb.2021.735416 PMC8595287 34804992

[B30] Millman A. Melamed S. Leavitt A. Doron S. Bernheim A. Hör J.. (2022). An expanded arsenal of immune systems that protect bacteria from phages. Cell Host Microbe 30 (11), 1556–1569.e1555. doi: 10.1016/j.chom.2022.09.017 36302390

[B31] Monson E. A. Trenerry A. M. Laws J. L. Mackenzie J. M. Helbig K. J. (2021). Lipid droplets and lipid mediators in viral infection and immunity. FEMS Microbiol. Rev. 45 (4), fuaa066. doi: 10.1093/femsre/fuaa066 33512504 PMC8371277

[B32] Morito D. Nishikawa K. Hoseki J. Kitamura A. Kotani Y. Kiso K.. (2014). Moyamoya disease-associated protein mysterin/RNF213 is a novel AAA+ ATPase, which dynamically changes its oligomeric state. Sci. Rep. 4, 4442. doi: 10.1038/srep04442 24658080 PMC3963067

[B33] Mukhopadhyay D. Sangaré L. O. Braun L. Hakimi M. A. Saeij J. P. (2020). Toxoplasma GRA15 limits parasite growth in IFNγ-activated fibroblasts through TRAF ubiquitin ligases. EMBO J. 39 (10), e103758. doi: 10.15252/embj.2019103758 32293748 PMC7232000

[B34] Noad J. von der Malsburg A. Pathe C. Michel M. A. KOmander D. Randow F. (2017). LUBAC-synthesized linear ubiquitin chains restrict cytosol-invading bacteria by activating autophagy and NF-κB. Nat. Microbiol. 2, 17063. doi: 10.1038/nmicrobiol.2017.63 28481331 PMC5576533

[B35] Otten E. G. Werner E. Crespillo-Casado A. Boyle K. B. Dharamdasani V. Pathe C.. (2021). Ubiquitylation of lipopolysaccharide by RNF213 during bacterial infection. Nature 594 (7861), 111–116. doi: 10.1038/s41586-021-03566-4 34012115 PMC7610904

[B36] Paumen M. B. Ishida Y. Muramatsu M. Yamamoto M. Honjo T. (1997). Inhibition of carnitine palmitoyltransferase I augments sphingolipid synthesis and palmitate-induced apoptosis. J. Biol. Chem. 272 (6), 3324–3329. doi: 10.1074/jbc.272.6.3324 9013572

[B37] Peng K. Liu R. Jia C. Wang Y. Jeong G. H. Zhou L.. (2021). Regulation of O-linked N-acetyl glucosamine transferase (OGT) through E6 stimulation of the ubiquitin ligase activity of E6AP. Int. J. Mol. Sci. 22 (19), 10286. doi: 10.3390/ijms221910286 34638625 PMC8508608

[B38] Perng Y. C. Lenschow D. J. (2018). ISG15 in antiviral immunity and beyond. Nat. Rev. Microbiol. 16 (7), 423–439. doi: 10.1038/s41579-018-0020-5 29769653 PMC7097117

[B39] Piccolis M. Bond L. M. Kampmann M. Pulimeno P. Chitraju C. Jayson C. B. K.. (2019). Probing the global cellular responses to lipotoxicity caused by saturated fatty acids. Mol. Cell 74 (1), 32–44.e38. doi: 10.1016/j.molcel.2019.01.036 30846318 PMC7696670

[B40] Pirbaluty A. M. Mehrban H. Kadkhodaei S. Ravash R. Oryan A. Ghaderi-Zefrehei M.. (2022). Network meta-analysis of chicken microarray data following avian influenza challenge-A comparison of highly and lowly pathogenic strains. Genes (Basel) 13 (3), 435. doi: 10.3390/genes13030435 35327988 PMC8953847

[B41] Pollaci G. Gorla G. Potenza A. Carrozzini T. Canavero I. Bersano A.. (2022). Novel multifaceted roles for RNF213 protein. Int. J. Mol. Sci. 23 (9), 4492. doi: 10.3390/ijms23094492 35562882 PMC9099590

[B42] Radoshevich L. Impens F. Ribet D. Quereda J. J. Nam Tham T. Nahori M. A.. (2015). ISG15 counteracts Listeria monocytogenes infection. Elife 4, e06848. doi: 10.7554/eLife.06848 26259872 PMC4530601

[B43] Rieser E. Cordier S. M. Walczak H. (2013). Linear ubiquitination: a newly discovered regulator of cell signalling. Trends Biochem. Sci. 38 (2), 94–102. doi: 10.1016/j.tibs.2012.11.007 23333406

[B44] Sugihara M. Morito D. Ainuki S. HIrano Y. Ogino K. Kitamura A.. (2019). The AAA+ ATPase/ubiquitin ligase mysterin stabilizes cytoplasmic lipid droplets. J. Cell Biol. 218 (3), 949–960. doi: 10.1083/jcb.201712120 30705059 PMC6400562

[B45] Takamatsu Y. Higashimoto K. Maeda T. Kawashima M. Matsuo M. Abe T.. (2017). Differences in the genotype frequency of the RNF213 variant in patients with familial moyamoya disease in Kyushu, Japan. Neurol. Med. Chir (Tokyo) 57 (11), 607–611. doi: 10.2176/nmc.oa.2017-0036 28931766 PMC5709714

[B46] Tashiro R. Niizuma K. Kasamatsu J. Okuyama Y. Rashad S. Kikuchi A.. (2021). Dysregulation of Rnf 213 gene contributes to T cell response via antigen uptake, processing, and presentation. J. Cell Physiol. 236 (11), 7554–7564. doi: 10.1002/jcp.30396 33973242

[B47] Thery F. Martina L. Asselman C. Zhang Y. Vessely M. Repo H.. (2021). Ring finger protein 213 assembles into a sensor for ISGylated proteins with antimicrobial activity. Nat. Commun. 12 (1), 5772. doi: 10.1038/s41467-021-26061-w 34599178 PMC8486878

[B48] Tian H. Yu K. He L. Xu H. Han C. Zhang X.. (2023). RNF213 modulates γ-herpesvirus infection and reactivation via targeting the viral Replication and Transcription Activator. Proc. Natl. Acad. Sci. U.S.A. 120 (12), e2218825120. doi: 10.1073/pnas.2218825120 36917666 PMC10041092

[B49] Tokunaga F. (2013). Linear ubiquitination-mediated NF-κB regulation and its related disorders. J. Biochem. 154 (4), 313–323. doi: 10.1093/jb/mvt079 23969028

[B50] Walsh S. C. Reitano J. R. Dickinson M. S. Kutsch M. Hernandez D. Barnes A. B.. (2022). The bacterial effector GarD shields Chlamydia trachomatis inclusions from RNF213-mediated ubiquitylation and destruction. Cell Host Microbe 30 (12), 1671–1684.e1679. doi: 10.1016/j.chom.2022.08.008 36084633 PMC9772000

[B51] Wilkinson K. D. Audhya T. K. (1981). Stimulation of ATP-dependent proteolysis requires ubiquitin with the COOH-terminal sequence Arg-Gly-Gly. J. Biol. Chem. 256 (17), 9235–9241. doi: 10.1016/S0021-9258(19)52535-2 6267067

[B52] Winkler G. S. Timmers H. T. (2005). Structure-based approaches to create new E2-E3 enzyme pairs. Methods Enzymol. 399, 355–366. doi: 10.1016/s0076-6879(05)99024-1 16338368

[B53] Yuan W. Krug R. M. (2001). Influenza B virus NS1 protein inhibits conjugation of the interferon (IFN)-induced ubiquitin-like ISG15 protein. EMBO J. 20 (3), 362–371. doi: 10.1093/emboj/20.3.362 11157743 PMC133459

[B54] Zheng N. Shabek N. (2017). Ubiquitin ligases: structure, function, and regulation. Annu. Rev. Biochem. 86, 129–157. doi: 10.1146/annurev-biochem-060815-014922 28375744

